# Supplementary material for: Content-rich biological network constructed by mining PubMed abstracts
Source: BMC Bioinformatics. 2004 Oct 8;5:147. doi: 10.1186/1471-2105-5-147 (PMC528731; doi:10.1186/1471-2105-5-147)
Supplement: Additional File 2 — The original results of the above study (non-essential files are deleted to keep the file size under the limit set by BMC bioinformatics). [file 1471-2105-5-147-S2.bz2 › chilibotAdditionalFile2/dip20/538ID11085509E10890/html/AR_BRCA1.html]

 


 **AR** and **BRCA1** 
  
Found 28 abstracts in PubMed, retrieved 20.  
 

 What does Google say? 
 PDF only 
| .edu only 

---

**Interactive relationship** (e.g. stimulation, inhibition, etc)

**Stimulatory relationship**- In transfected prostate and breast cancer cell lines,  **BRCA1**  enhanced  **AR**  dependent transactivation of a probasin derived reporter gene.  Ref: 11085509 Cancer Res, 2000
- Here, we report that  **BRCA1**  interacts with androgen receptor  [ **AR** ]   **AR**  and enhances  **AR**  target genes, such as p21 WAF1 CIP1, that may result in the increase of androgen induced cell death in prostate cancer cells.  Ref: 11016951 Proc Natl Acad Sci U S A, 2000
- The  **BRCA1**  enhanced  **AR**  transactivation can be further induced synergistically with  **AR**  coregulators, such as CBP, ARA55, and ARA70.  Ref: 11016951 Proc Natl Acad Sci U S A, 2000
- On the contrary, it was proposed that a longer CAG repeat sequence might be associated with an increased risk of breast cancer in  **BRCA1**  mutation carriers, thus suggesting a different role of the  **AR**  CAG polymorphism in sporadic and inherited breast cancers.  Ref: 11368874 Cancer Lett, 2001
**Neutral relationship**- These findings suggest that  **BRCA1**  may directly modulate  **AR**  signaling and, therefore, may have implications regarding the proliferation of normal and malignant androgen regulated tissues.  Ref: 11085509 Cancer Res, 2000
- RESULTS The  **AR**  was only present in 12% of  **BRCA1**  mutated tumors, with mutations located at the C terminal half of the  **BRCA1**  gene.  Ref: 12779088 Breast Cancer Res Treat, 2003
- The CAG repeat within exon 1 of the androgen receptor  [ **AR** ]   **AR**  gene is reportedly associated with breast cancer phenotype in  **BRCA1**  mutation carriers.  Ref: 12404104 Eur J Hum Genet, 2002
- Androgen receptor  [ **AR** ]  CAG repeat length in Jewish Israeli women who are  **BRCA1**  2 mutation carriers association with breast ovarian cancer phenotype.  Ref: 12404104 Eur J Hum Genet, 2002
- In addition,  **BRCA1**  was shown to interact physically with both the  **AR**  and the p160 coactivator, glucocorticoid receptor interacting protein 1.  Ref: 11085509 Cancer Res, 2000
- Two hundred and twenty seven  **BRCA1**  2 mutation carriers were genotyped for the polymorphic  **AR**  CAG repeat, and allele size was correlated with breast ovarian cancer morbidity parameters.  Ref: 12404104 Eur J Hum Genet, 2002
- The CAG repeat in exon 1 of the androgen receptor  [ **AR** ]   **AR**  genes has been postulated as both a susceptibility allele and phenotypic modifier in  **BRCA1**  associated breast cancers.  Ref: 10717532 Eur J Cancer, 2000
- These data suggest that  **AR**  allele length affects age of diagnosis of ovarian cancer, irrespective of BRCA  [ **BRCA1** ]  mutation status.  Ref: 11221880 Cancer Res, 2001
- The loss of the  **AR**  expression, as shown by immunohistochemistry, together with the observed loss of other steroid hormone receptors in  **BRCA1**  mutated tumors may lead to a hormone independent growth or to anti hormone resistant growth of these tumors.  Ref: 12779088 Breast Cancer Res Treat, 2003

**Non-interactive relationship** (e.g. studied together, co-existance, homology, etc.)

- Using a case series study design, 179 consecutive Ashkenazi Jewish ovarian cancer patients were genotyped for  **AR**  repeat length and BRCA  [ **BRCA1** ]  mutation status.  Ref: 11221880 Cancer Res, 2001
- Our objectives were to test whether polymorphic variation in the CAG n repeat of the androgen receptor  [ **AR** ]   **AR**  gene affects penetrance of germ line BRCA  [ **BRCA1** ]  mutations for ovarian cancer or age of diagnosis for ovarian cancer.  Ref: 11221880 Cancer Res, 2001
- Modification of  **BRCA1**  associated breast cancer risk by the polymorphic androgen receptor  [ **AR** ]  CAG repeat.  Ref: 10205268 Am J Hum Genet, 1999
- Cotransfection of p160 coactivators markedly potentiated  **BRCA1**  mediated enhancement of  **AR**  signaling.  Ref: 11085509 Cancer Res, 2000
- In the present study, the role of  **BRCA1**  in ligand dependent androgen receptor  [ **AR** ]   **AR**  signaling was assessed.  Ref: 11085509 Cancer Res, 2000
- No effect on survival was seen for polymorphisms in ATM,  **BRCA1**  2, CHK2, KU70, NBS1, RAD51, RAD52, XRCC3,  **AR** , COMT, NQO1, VDR, ADH3, CYP1A1, GSTP1, TGF beta, or CDH1.  Ref: 12036913 Cancer Res, 2002
- Since this growth factor is induced by androgens, we studied the androgen receptor  [ **AR** ]   **AR**  expression in BRCA  [ **BRCA1** ]  mutated tumors and in matched sporadic breast tumors.  Ref: 12779088 Breast Cancer Res Treat, 2003
- There was no association between  **AR**  repeat length and presence of a BRCA  [ **BRCA1** ]  mutation.  Ref: 11221880 Cancer Res, 2001
- We genotyped 188  **BRCA1**  2 carriers 122 affected and 66 unaffected with breast cancer, 158 of them of Ashkenazi origin, 166 BC cases without  **BRCA1**  2 mutations and 156 Ashkenazi control individuals aged over 56 for the  **AR**  CAG and GGC repeats.  Ref: 11437399 Br J Cancer, 2001
- We have extended our LOH analysis of chromosome 3p to include 102 ovarian tumor specimens 29 and 73 samples were previously examined for LOH of chromosome 3p and 17 markers, respectively, using additional polymorphic markers, to assess the coordinate LOH of loci representing the three chromosome 3p minimal regions of deletions von Hippel Lindau syndrome VHL, thyroid hormone receptor beta, and fragile histidine triad FHIT and LOH of other important loci tumor protein 53 TP53, breast cancer 1 early onset  [ **BRCA1** ]   **BRCA1** , breast cancer 2 early onset, retinoblastoma 1, ornithine carbamoyltransferase, and androgen receptor  [ **AR** ]  or somatic mutations in TP53.  Ref: 12112314 Mol Carcinog, 2002
- BRCA2 not only associates with  **AR**  and GRIP1 but also cooperates with both the histone acetyltransferase P CAF and  **BRCA1**  to enhance  **AR**  and GRIP1 mediated transactivation.  Ref: 12756300 Proc Natl Acad Sci U S A< MedlineTA>Proc Natl Acad Sci U S A, 2002
- Using a sample of women who inherited germline  **BRCA1**  mutations, we compared  **AR**  CAG repeat length in 165 women with and 139 women without breast cancer.  Ref: 10205268 Am J Hum Genet, 1999
- We conclude that, in contrast to previous observations, if there is any effect of the  **AR**  repeat length on  **BRCA1**  penetrance, it is likely to be weak.  Ref: 11437399 Br J Cancer, 2001
- METHODS Paraffin embedded breast tumors of carriers of a  **BRCA1**  mutation n=41, median age of patients at time of surgery was 41 years range 28 59 years or a BRCA2 mutation n=14, median age 41 years range 31 85 years were analyzed for the presence of ER alpha, PR, P53 and  **AR**  using standard immunohistochemical techniques.  Ref: 12779088 Breast Cancer Res Treat, 2003
- However, ovarian cancer patients from both groups with or without BRCA  [ **BRCA1** ]  mutation who carried a short  **AR**  allele were diagnosed an average of 7.2 95% confidence interval, 2.3 12.1 years earlier than patients who did not carry a short allele P = 0.004.  Ref: 11221880 Cancer Res, 2001
- Our results support the hypothesis that age at breast cancer diagnosis is earlier among  **BRCA1**  mutation carriers who carry very long  **AR**  CAG repeats.  Ref: 10205268 Am J Hum Genet, 1999
- A previous study has suggested that  **BRCA1**  carriers with longer lengths of the CAG repeat in the androgen receptor  [ **AR** ]   **AR**  gene are at increased risk of breast cancer BC.  Ref: 11437399 Br J Cancer, 2001
- Nine of 11  **AR**  informative carriers of germline  **BRCA1**  mutations demonstrated nonrandom X chromosome inactivation.  Ref: 10050867 J Natl Cancer Inst, 1999
- Increase of androgen induced cell death and androgen receptor  [ **AR** ]  transactivation by  **BRCA1**  in prostate cancer cells.  Ref: 11016951 Proc Natl Acad Sci U S A, 2000
- Together, these data suggest that the  **BRCA1**  may function as an  **AR**  coregulator and play positive roles in androgen induced cell death in prostate cancer cells and other androgen  **AR**  target organs.  Ref: 11016951 Proc Natl Acad Sci U S A, 2000
- There is no conclusive evidence of association between  **AR**  CAG repeat size and breast or ovarian cancer risk in Jewish  **BRCA1**  2 mutation carriers.  Ref: 12404104 Eur J Hum Genet, 2002
- CAG and GGC repeat polymorphisms in the androgen receptor  [ **AR** ]  gene and breast cancer susceptibility in  **BRCA1**  2 carriers and non carriers.  Ref: 11437399 Br J Cancer, 2001
- Also, we obtained high success rates in determining the number of CAG repeats in the androgen receptor  [ **AR** ]  gene, characterizing tetranucleotide microsatellites in six gene loci, and screening for mutations in the  **BRCA1**  2 genes in a subset of phenol chloroform DNA extracts.  Ref: 11401920 Cancer Epidemiol Biomarkers Prev, 2001
